# Determinants of Glycemic Control among Insulin Treated Diabetic Patients in Southwest Ethiopia: Hospital Based Cross Sectional Study

**DOI:** 10.1371/journal.pone.0061759

**Published:** 2013-04-19

**Authors:** Mulugeta Tarekegn Angamo, Belete Habte Melese, Wubeante Yenet Ayen

**Affiliations:** 1 Department of Pharmacy, Jimma University, Jimma, Oromiya, Ethiopia; 2 Department of Internal Medicine, Jimma University, Jimma, Oromiya, Ethiopia; College of Pharmacy, University of Florida, United States of America

## Abstract

**Background:**

Good glycemic control reduces the risk of diabetic complications. Despite this, achieving good glycemic control remains a challenge in diabetic patients. The objective of this study is to identify determinants of glycemic control among insulin treated diabetic patients at Jimma University Hospital, Southwest Ethiopia.

**Methods:**

Hospital-based cross-sectional study was conducted on systematically sampled 284 insulin-treated diabetic patients with a regular follow up. Data was collected by interviewing patients during hospital visits and reviewing respective databases of September 2010 to December 2011. Data collection took place from February 20 to May 20, 2012. Poor glycemic control was defined as fasting blood sugar (FBS) ≥126 mg/dL. Binary logistic regression analysis was conducted to identify predictors of poor glycemic control.

**Results:**

Patients had a mean age of 41.37 (±15.08) years, 58.5% were males, the mean duration of insulin treatment was 4.9 (±5.1) years, 18.3% achieved good glycemic control (FBS≤126 mg/dL), 95% self-reported repeated use of disposable insulin syringe-needle and 48% correctly rotating insulin injection sites. Most (83.1%) of study participants had one or more complications. On multivariable logistic regression analyses, body weight of >70 Kg (AOR = 0.21; P<0.001), total daily dose of insulin ≤35 IU/day (AOR = 0.26; P<0.001), total daily dose variation without checking glycemic level (AOR = 3.39; P = 0.020), knowledge deficit about signs and symptoms of hyperglycemia (AOR = 3.60; P = 0.004), and non-adherence to dietary management (AOR = 0.35; P = 0.005) were independent predictors of poor glycemic control.

**Conclusions:**

The proportion of patients with poor glycemic control was high, which resulted in the development of one or more complications regardless of duration on insulin treatment. Hence, appropriate management of patients focusing on the relevant associated factors and independent predictors of poor glycemic control would be of great benefit in glycemic control.

## Introduction

Diabetes mellitus is a chronic disease with a high prevalence and a growing concern worldwide. As per International Diabetes Federation fifth edition (2012) update, more than 371 million people have diabetes in the world and now a day the burden is increasing particularly in developing countries. About 80% of diabetes deaths occur in low and middle income countries. Ethiopia with national prevalence of 3.36%, 23,869 diabetes related deaths and with mean 25 USD diabetes related expenditure per person is highly affected. There is no cure for this disease and it requires continuing medical care and education to prevent acute complications and to reduce the risk of long-term complications. Poor glycemic control is the most common cause of hospital admissions and complications in diabetes [1], [2]. Evidences show that maintaining good glycemic control is main therapeutic goal for all patients with diabetes to prevent organ damage and other microvascular and macrovascular complications. Most national bodies have recommended good glycemic control with fasting blood sugar (FBS) level from 70 to 130 mg/dL. Glycemic control, however, is not an easy task for many patients. It is well known that even in clinical trials, and routinely in clinical practice, the majority of patients fail to achieve good glycemic control [3].

Different studies in systematic review showed that good glycemic control is achieved in less than 50% of diabetic patients. The reasons for this failure are complex and multifactorial of which both patient and healthcare provider related factors may contribute to poor glycemic control [4], [5]. However, proving exactly what factors lead to the loss of glycemic control can be challenging. There have been some investigations in this area. Studies with type I diabetic patients have found correlations between poor glycemic control and factors such as socio-demographic characteristics; insulin therapy knowledge and skill deficit; poor adherence to insulin regimen, self-care, exercise and dietary plan combined with poor interaction between the patient and health care providers [6], [7].

Significant knowledge and skill deficits have been found among 50–80% of diabetic patients who failed to achieve good glycemic control [8]. In a survey of 164 College students with type I diabetes in the USA, the most commonly reported barriers to effective glycemic control included diet selection, irregular insulin injection schedules, alcohol use, fear of hypoglycemia, and finance. Factors associated with improved control were increased sense of responsibility for self-care, increased frequency of blood glucose testing, regular exercise and contact with healthcare providers, and fear of hyperglycemia [9]. A study conducted on the assessment of the patterns of diabetic complications in Jimma University Hospital showed that the frequency of chronic complications was high, which is associated with poor diabetes care at the hospital [10], [11]. Therefore, this study was conducted to identify risk factors, if not managed appropriately, could contribute to poor glycemic control and subsequent chronic complications among insulin treated diabetic patients at Jimma University Hospital, Southwest Ethiopia.

## Participants and Methods

### Study setting and period

This study was conducted from February 20 to May 20, 2012 at Diabetes Clinic, Jimma University Hospital. Jimma University Hospital is the only teaching and referral hospital located in Jimma Town, Southwest Ethiopia. It provides services for approximately 9000 inpatient and 80,000 outpatient attendances a year from the catchment population of about 15 million people. It has bed capacity of 450 and 750 staffs of both supportive and professional. Diabetes Clinic is one of the chronic follow up clinics of the hospital providing services from Monday to Friday. Patients are evaluated by a multidisciplinary team of internists, medical residents, senior medical students, clinical pharmacists, and general nurses at baseline and during their visits.

### Study design and data collection

Hospital based cross sectional study was conducted to examine the role of socio-demographic, anthropometric, clinical and other relevant characteristics in glycemic control among systematically sampled 284 insulin treated diabetic patients at Jimma University Hospital, Southwest Ethiopia. Data was retrieved through face-to-face interview of patients and review of respective database of September 2010 to December 2011. Face-to-face interview of the patient about self care practices and barriers to insulin therapy, storage condition of insulin, injection site rotation, single use disposable syringe-needle change practice, identification and management of side effects of insulin was conducted by trained interviewer. Moreover, observation of injection sites, average patient consultation time, insulin injection process demonstration (re-suspending, withdrawing, measuring, injecting) by using validated check lists was recorded. Patient chart reviewing for determining of average FBS level, weight, height, and total daily insulin dose for at least 4 visits or measurements and for identification of complications was carried out.

### Participant eligibility criteria

For the purpose of this study, patients with type I and type II diabetes mellitus treated with insulin were selected that reduced the population size from 2,336 to 1019. To avoid bias and ensure that the study participants were actively following the diabetic clinic, the inclusion criteria were established. Patients with regular follow up and at least 4 measurements of fasting blood sugar (FBS) level in the past year, patients only on insulin regimen, age greater than or equal to 15 years, and those who can inject insulin by themselves were included in the study. Based on the inclusion criteria and systematic sampling technique, out of 1019 insulin treated diabetic patients in the database, 284 were included in the study by considering confidence level of 95% with margin of error 5%, and response distribution of 50%. Patients with the following conditions were excluded: hospitalized and/or with psychiatric disorder during the data collection time (since there is self care assessment and questionnaire investigation), those who are not willing to participate and not signing the written informed consent.

### Measures and operational definitions

Independent variables and outcomes measures are defined as follow.

#### Socio-demographic and behavioural factors

Socio-demographic variables, such as gender, age, education, marital status and occupation were recorded using checklist. Behavioural factors such as current cigarette smoking and alcohol consumption were defined as follow. Participants, with regard to their smoking habit, were categorized as (a) nonsmokers, if they had never smoked or quit smoking just a year before; (b) smokers, if they regularly smoke at least one cigarette daily. Alcohol consumption was assessed by asking participant to report frequency of alcohol intake, accordingly at least twice weekly of any alcoholic drinks consumed was considered as alcohol consumer for the purpose of this analysis.

#### Anthropometric and Clinical measures

Anthropometric data such as weight, height, and body mass index (BMI) were recorded on the prepared checklist. Based on BMI, participants were grouped into different categories as normal range (BMI<25 kg/m^2^), and overweight and obese (BMI≥25 kg/m^2^). Clinical measures including duration of disease/duration on insulin treatment, measurements of FBS level were abstracted from patients' database. The participants with FBS <126 mg/dL were categorized as good glycemic control and those with FBS of ≥126 mg/dL were categorized as poor glycemic control as per American Diabetic Associations (ADA) recommendations [12]. Lipohypertrophy/lipoatrophy was measured as the presence of one or more scar and/or nodules or localized loss of fat tissue at the insulin injection sites after the start of insulin injection, respectively, which had been diagnosed by medical doctors working in the diabetic follow up clinic during data collection period.

#### Patient's knowledge, skill and adherence about insulin therapy

For the assessment of knowledge about signs and symptoms of hyperglycemia, a set of questions consisting of 12 knowledge and skill parameters were structured and the participants were interviewed face-to-face. For the correct response to knowledge and skill assessment, a score of 1 was given, while for incorrect response it was given zero. The scores of each response were added and converted into a percent score. For the purpose of this study, the responses with higher than 60% scores were considered satisfactory, while below 60% were considered unsatisfactory. Patients' adherence to insulin regimen, self-care and life style modification as recommended by health professionals were measured by using 16 items designed by experts' opinions from different literatures and measures were collected through self-report and observation. If a participant is able to achieve 85% and above the participant is categorized as adherent; score of 50% to 84.9% as partially adherent and less than 50% as non-adherent. Rotation of injection sites was defined as injecting insulin by rotating within the same anatomic region with the same trend and using another anatomical area for the next injection (i.e. for one week period rotating in the arms and for another week rotating at the thigh, etc), but not morning injection within one region and evening in another.

#### Insulin storage condition and use of syringe-needle

Regarding the storage condition of insulin, acceptable or appropriate insulin storage conditions are defined when insulin is stored in the functioning refrigerator at 2–8°C. Storing insulin in a water proof container like plastic bag and put into a clay pot filled half way with water or in a container wrapped with rope, which is kept in moist area was considered as fair; if not meeting the above two storage conditions, it was categorized as poor storage (storing in cabinet, box, and bag). Repeated use of disposable insulin syringe-needle was defined as reuse of disposable syringe-needle more than 3 injections per needle.

#### Consultation time

For the purpose of this study adequate time for consultation is defined as if the time spent between the patient and physician discussing regarding diabetes prognosis and medication use during refilling appointment is greater than or equal to 10 minutes, which is considered as adequate time.

### Data analysis

Data entry and analysis was carried out using Statistical Product and Service Solution software (version 16.0 for windows; SPSS). Data is reported using mean (±S.D.) for continuous variables and proportions for categorical variables. Chi-square test was used to assess statistical significance of the difference in the percentages of good and poor glycemic control according to independent categorical variables. Binary logistic regression analysis was conducted to identify factors, if not managed appropriately, could lead to poor glycemic control and subsequent complications. Statistical significance was set at p<0.05.

### Ethical statement

This study was approved by the Institutional Review Board, Jimma University. Patient's written informed consent to participate in the study was obtained after comprehensive explanation of the purpose and procedure of the study. Patients were informed about their rights to refuse or withdraw, and about confidentiality of the individual information obtained. During data collection process, patients at any risk of complication, or using insulin therapy wrongly and inappropriately were told to correct at spot after the response was taken.

## Results

### Socio-demographic characteristics

This study included 284 participants, of which 58.5% were males. The median age of participants was 40.00 (16–92) years and most (81.4%) of the participants were ≤55 years old. About 30.6% participants were illiterate and 17.6% completed college and above. More than half (65.1%) of the participants were married, followed by single (22.9%). Thirty one-percents of patients were non-employed; while the rest are farmers, merchants, employee, and students. Three-fourth (75%) of the participants' body weight was <70 Kg. About 69.4% of the participants' body mass index was normal (<25 kg/m^2^) followed by 30.6% overweight and obese (≥25 kg/m^2^) (Table 1).

**Table 1:** Demographic characteristics and glycemic control of patients receiving insulin at Jimma University Hospital, Southwest Ethiopia.

Variables	Good glycemic control, n (%) (FBS <126 mg/dL), N = 52	Poor glycemic control, n (%) (FBS ≥126 mg/dL), N = 232
Age of the participants		
• 15–25	5(9.6)	45(19.4)
• 26–35	8(15.4)	55(23.7)
• 36–45	20(38.5)	37(15.9)
• 46–55	12(23.1)	49(21.1)
• >/ = 56	7(13.5)	46(19.8)
Gender		
• Male	32(61.5)	134(57.8)
• Female	20(38.5)	98(42.2)
Educational status		
• Illiterate	15(28.8)	72(31.0)
• 1–4 class	7(13.5)	27(11.6)
• 5–8 class	11(21.2)	49(21.1)
• 9–10 class	3(5.8)	26(11.2)
• 11–12 class	4(7.7)	20(8.6)
• College and above	12(23.1)	38(16.4)
Marital status		
• Not married	10(19.2)	55(23.7)
• Married	33(63.5)	152(65.5)
• Widowed/Divorced	9(17.3)	25(10.7)
Occupational status		
• Merchant	5(9.6)	18(7.8)
• Farmer	17(32.7)	56(24.1)
• Employed	16(30.8)	49(21.1)
• Non-employed	13(25.0)	77(33.2)
• Student	1(1.9)	32(13.8)
Body weight		
• <70 Kg	29(55.8)	184(79.3)
• ≥70 Kg	23(44.2)	48(20.7)
Body mass index		
• <25 Kg/m^2^	30 (57.7)	167(72.0%)
• ≥25 Kg/m^2^	22(42.3)	65(28.0%)
Type of Diabetes		
• Type 1	32(61.5)	131(56.5)
• Type 2	20(38.5)	101(43.5)

**Note:** Good glycemic control is defined as a fasting blood glucose level of <126 mg/dl, based on American Diabetes Association criteria [12].

### Glycemic control

Of 284 participants, 52 (18.3%) had good glycemic control, while significant proportion of patients, 232 (81.7%) had poor glycemic control. Overall, the mean value of FBS for the whole sample was 163.2 mg/dL (SD = 45). Unadjusted logistic regression analysis showed that diabetes was more likely to be poorly controlled (FBS level ≥126 mg/dL) among those with younger age group of 15–25 (COR = 1 (reference), P = 0.007) and middle age group, 36–45 (COR = 0.21, 95% CI = 0.07–0.60, P = 0.004); body weight >70 kg (COR = 0.33, 95% CI = 0.18–0.62, P = 0.001); body mass index ≥25 Kg/m^2^ (COR = 0.53, CI = 0.29–0.99, P = 0.045); injection of lower (≤35 IU/day) daily dose of insulin (COR = 0.26,95% CI = 0.14–0.49, P<0.001); variation of daily dose of insulin without checking blood glucose level (COR = 2.74, 95% CI = 1.11–6.72, P = 0.028) (Table 2); presence of complication(s) (COR = 0.39, 95% CI = 0.16–0.98, P = 0.043); non-adherence to diabetic dietary plan of more vegetables and fruits (COR = 0.33, 95% CI = 0.17–0.64, P = 0.001) and knowledge deficit about signs and symptoms of hyperglycemia (COR = 2.47, 95% CI = 1.14–5.32, P = 0.021) as shown in Table 3.

**Table 2:** Bivariate logistic regression analysis of factors affecting glycemic control at Jimma University Hospital, Southwest Ethiopia.

Variables	N (%)	COR (95%CI)	P-value
Age of the participants			
15–25	50(17.6)	1.0	0.007
26–35	63(22.2)	0.76(0.23,2.49)	0.656
36–45	57(20.1)	0.21(0.07,0.60)	0.004
46–55	61(21.5)	0.45(0.15,1.39)	0.166
≥56	53(18.7)	0.73(0.22, 2.47)	0.613
Gender:			
Male	166(58.5)	0.86(0.46,1.58)	0.617
Female	118(41.5)	1.0	
Educational status			
Illiterate	87(30.6)	1.0	0.781
1–4 class	34(12.0)	0.80(0.30, 2.19)	0.668
5–8 class	60(21.1)	0.93(0.39, 2.19)	0.865
9–10 class	29(10.2)	1.81(0.48, 6.75)	0.380
11–12 class	24(8.5)	1.04(0.31, 3.49)	0.947
College and above	50(17.6)	0.66(0.28, 1.55)	0.340
Marital status			
Single	65(22.9)	1.0	0.523
Married	185(65.1)	0.84(0.39,1.81)	0.652
Widowed	24(8.5)	0.44(0.15,1.34)	0.148
Divorced	10(3.5)	0.73(0.13,3.94)	0.712
Average body weight (Kg)			
≤70	213(75.0)	1.0	
>70	71(25.0)	0.33(0.18,0.62)	0.001
Average body mass index (Kg/m2)			
<25 (normal weight)	197(69.4)	1.0	
≥25(overweight & obesity)	87(30.6)	0.53(0.29–0.99)	0.045
Types of DM			0.504
Type I	163(57.4)	1.0	
Type II	121(42.6)	1.23(0.67–2.28)	
Total daily dose of insulin in IU/day			
≤35 IU	100(35.2)	0.26(0.14, 0.49)	0.000
>35 IU	184(64.8)	1.0	
Variation of daily dose of insulin			
Yes	67(23.6)	2.74(1.11,6.72)	0.028
No	217(76.4)	1.0	
Injection of insulin at lipohypertrophied area			
Yes	45(15.8)	1.26(0.53,3.00)	0.603
No	239(84.2)	1.0	

**Table 3:** Bivariate logistic regression analysis of factors affecting glycemic control at Jimma University Hospital, Southwest Ethiopia.

Variables	N (%)	COR (95%CI)	P-value
Rotation of injection sites			
Yes	136(47.9)	1.0	
No	148(52.1)	1.01(0.55,1.84)	0.976
Duration on insulin treatment (Years)			
≤5 years	192(67.6)	1.0	0.182
5–10 years	54(19.0)	1.32(0.59,2.92)	0.499
>10 years	38(13.4)	3.07(0.89,10.50)	0.074
Frequency of blood glucose testing habit at home			
Never	265(93.3)	1.0	0.948
2–4 times in a week	5(1.8)	0.89(0.10,8.09)	0.914
Sometimes as needed	14(4.9)	0.81(0.22,3.02)	0.755
Knowledge about of insulin therapy			
Satisfactory	122(43.0)	0.88(0.48,1.62)	0.678
Unsatisfactory	162(57.0)	1.0	
Knowledge about sign & symptom of hyperglycemia			
Yes	196(69.0)	1.0	
No	88(31.0)	2.47(1.14, 5.32)	0.021
Adherence to eat vegetables and fruit in daily meal			
Yes	137(48.2)	1.0	
No	147(51.8)	0.33(0.17, 0.64)	
Adherence to insulin regimen, self-care and life style			
Adherent	24(8.5)	1.0	0.509
Partially adherent	168(59.2)	1.42(0.52,3.86)	0.495
Non-adherent	92(32.4)	1.86(0.63,5.50)	0.263
Presence of complication			
One complication	55(19.4)	0.39(0.16,0.98)	0.043
Two complications	70(24.6)	1.01(0.40,2.53)	0.046
Three or more complications	111(39.1)	0.98(0.33,2.99)	0.982
No complication	48(16.9)	1.0	0.996
Insulin storage condition			
Good	118(41.5)	1.0	0.753
Fair	126(44.4)	1.28(0.67,2.44)	0.460
Poor	40(14.1)	1.20(0.48,3.05)	0.696
Average consultation time			
Inadequate	239(84.2)	1.14(0.51,2.54)	0.749
Adequate	45(15.8)	1.0	
Currently smoke cigarette			
Yes	9(3.2)	1.82(0.22–0.14.88)	0.576
No	275(96.8)	1.0	
Currently consume alcohol			
Yes	84(29.6)	1.32(0.67,2.63)	0.425
No	200(70.4)	1.0	

**COR**: Crude odds ratio.

However, other factors such as gender, educational status, type of diabetes (Table 2) alcohol drinking, cigarette smoking, and duration on insulin treatment (Table 3) did not show correlation with poor glycemic control. In the present study alcohol intake and cigarette smoking were not identified as risk factors, a finding that can be explained as most of the patients use to drink alcohol or smoke cigarette for refreshment habit and moderate alcohol consumption has been reported to enhance insulin sensitivity and improve glycemic control [13]. Other studies showed that cigarette smoking by diabetic patients is associated with an increased prevalence of microvascular complications, at least partly mediated through poor glycemic control [14].

### Multivariate analysis of factors associated with poor glycemic control

Adjusted multivariate logistic analysis was performed to identify independent predictors of glycemic control among insulin treated diabetic patients. For the purpose of this analysis, variables identified with p-value <0.05 by bivariate analysis were used for multivariate analysis. Accordingly, in the multivariate logistic analysis diabetes was more likely to be poorly controlled among those with body weight of >70 kg (AOR = 0.21, 95% CI = 0.10–0.45, P<0.001), total daily dose of insulin ≤35 IU/day (AOR = 0.26, 95% CI = 0.13–0.54, P<0.001), daily insulin dose variation without checking blood glucose level (AOR = 3.39, 95% CI = 1.21–9.50, P = 0.020), knowledge deficit about signs and symptoms of hyperglycemia (AOR = 3.60, 95% CI = 1.51–8.55, P = 0.004), and non-adherence to diabetic meal plan of more vegetables and fruits in daily meal (AOR = 0.35, 95% CI = 0.17–0.73, P = 0.005). This analysis indicated that participants with body weight >70 Kg were 0.21 times less likely to have good glycemic control as compared to those with body weight of ≤70 kg. Compared to participants who were taking daily dose of >35 IU/day insulin, those taking ≤35 IU/day insulin were 0.26 times less likely to have good glycemic control. Participants who did vary total daily dose of insulin without checking blood glucose level were 3.39 times more likely to have poor glycemic control as compared to those who did not vary total daily dose. Similarly, regarding participants' knowledge about hyperglycemia, those participants with knowledge deficit about signs and symptoms of hyperglycemia were 3.60 times more likely to have poor glycemic control. Participants who were non-adherent to eat more vegetables and fruit in daily meal were 0.35 times less likely to have good glycemic control as compared to those who adhered to eat more vegetables and fruits in each daily meal (Table 4). However, other factors were not significant to independently predict the poor glycemic control among insulin treated diabetic patients.

**Table 4:** Multivariate logistic regression analyses predicting poor glycemic control at Jimma University Hospital, Southwest Ethiopia.

Independent predictors of poor glycemic control	B	AOR (95.0% CI)	P-value
Body Weight			
≤70 kg			
>70 kg	−1.56	0.21 (0.10,0.45)	<0.001
Total daily dose of insulin in IU/day			
≤35 IU	−1.33	0.26(0.13,0.54)	<0.001
>35 IU		1.0	
Variation of daily dose of insulin without checking of glycemic level:			
Yes		3.39(1.21,9.50)	
No	1.22	1.0	0.020
Knowledge about signs and symptoms of hyperglycemia			
Yes		1.0	
No	1.28	3.60 (1.51,8.55)	0.004
Adherence to eat more vegetables and fruit in daily meal			
Yes		1.0	
No	−1.10	0.35(0.17,0. 73)	0.005

**B:** Beta, slope of logistic regression line; **AOR:** Adjusted odds ratio.

## Discussion

The corner stone in managing the diabetes mellitus is to achieve the glycemic control, which is essential for the prevention of short and long-term complications. Insulin is one of the treatment modalities and given either as single agent therapy for type I diabetic patients or as add-on therapy for type II diabetic patients who are not achieving the glycemic control by oral hypoglycemic agents. In the present study several important findings were obtained and poor glycemic control (FBS ≥126 mg/dL) was found in most of participants (81.7%) with only 18.3% of participants achieved good glycemic control (FBS <126 mg/dL) as per ADA recommendations [12], despite mean duration of insulin treatment is about 5 years. The results of this study suggest that greater effort is needed to improve glycemic control and treatment outcomes among patients treated with insulin at Jimma University Hospital. The questions of what predicts poor glycemic control has not been answered rationally in study area. In the present study, the possible factors related to poor glycemic control among most of the participants (81.7%) have been identified using multiple logistic regression analysis. The results obtained from multivariate logistic regression analysis of the present study revealed that higher body weight, lower total daily dose, total daily dose variation without evidence of blood glucose level, knowledge deficit about signs and symptoms of hyperglycemia, and poor adherence to dietary plan were independent predictors of poor glycemic control.

The present study showed that age of the participants had a significantly associated difference for young (P = 0.007) and middle age adults (P = 0.004), whereas adults aged ≥56 years were less likely to have poor glycemic control (P = 0.613), but generally age was not identified as independent predictor of glycemic control (P>0.05). The present finding is in consistent with that reported by other studies from USA [15] and China [16]. However, it is not in consistent with a findings from Jordan [17], [18] and Iran [19]. The present finding, which reported that younger and middle age group was associated with poor glycemic control can be explained that this age group of diabetic patients could be reluctant about the disease control, self-care and adherence to treatment recommendations due to busy life schedule and/or less interaction with health care providers. However, in this group of patients other barriers to achieving good glycemic control might exist that suggest future studies to explore this association.

Three-fourth of the participants' (75%) body weight was ≤70 kg and it was significantly associated (P = 0.001) with glycemic control and was identified as one of the independent predictors of poor glycemic control (AOR = 0.21, P<0.001) in the present study. This might be due to an increase in weight while height is constant results in overweight or obesity, which could lead to insulin resistances and ultimately poor glycemic control. Similar results were reported from India [20].

The appropriate insulin dosage is dependent on the glycemic response of the individual to food intake, exercise regimens and other life style managements. A dosage algorithm suited to the individual's needs and glycemic goals should be developed. In the present study, the lower total daily dose of insulin (≤35 IU/day) among study participants was significantly associated (P<0.001) with poor glycemic control and it was one of the independent predictors of poor glycemic control (AOR = 0.26, P<0.001). In the present study, compared to the Duke University Medical Centre, USA, guidelines' recommendation [21], more than one third of the participants were using smaller maintenance dose that might be the possible reason for the majority of the study participants' to be at elevated FBS level (poor glycemic control). The poor glycemic control is about twice as compared to the study in Pakistan (46.7%) [22], and still higher compared to the study in Jordan (65.1%) [17], Kuwait (66.7%) [23], and UK (69%) [24] reports. Possible reasons could be lack of awareness and most importantly lack of appropriate guidelines and diabetes education for both healthcare givers and patients in the study area.

Injection site rotation and disposing single use disposable syringe needles is an important component of insulin administration and is helpful in preventing lipodystrophy and achieving glycemic goal. In the present study, 52% of the patients did not rotate the insulin injection sites and 95% of patients re-use disposable syringe-needle five to seven days until it is no longer comfortable, which is beyond the recommended re-use (3 times). This might be due to inadequate patient education on injection sites rotation, reluctance to throw away used one, inadequate availability of disposable syringe-needles or poor economic status to afford for single use disposable syringe-needles. The present study finding showed that about 44% of the participants had lipohypertrophy at injection sites, a finding which is lower than findings reported from Turkey (48.7%) [25] and Egypt (54.5%) [26] and higher than the report (28.7%) from Germany [27], however both variables (failure to rotate injection sites and re-using disposable syringe needle) were not found as predictors of poor glycemic control. Rotation of injection sites and avoiding re-using of disposable syringe needles is critical to prevent lipohypertrophy. Lipohypertrophy occurs because patients inject the same site day after day using the same needle and re-used needles cause repeated trauma and contribute to the formation of lypohypertrophy [25], [28]. Lipohypertorphy, a complication of insulin injections has been linked to poor glycemic control as injecting into lipohypertrophied site can make insulin absorption erratic (25% absorption reduction from injection site) and affect overall blood glucose level [29], [30].

The complications of diabetes mellitus are far less common and less severe in people who have good glycemic control. In this study, 83% of the patients had at least one chronic complication, despite 67% of the patients were on insulin for shorter duration (≤5years). This is because majority of patients (81.7%) did not achieve good glycemic control and such complications might partly mediated through poor glycemic control. Among the complications, microvascular complications constitute the largest part followed by microvascular and macrovascular together. However, a study from China [31] and Greece [32] showed that at least one chronic complication was diagnosed in the study participants whose proportion was lower than the present study.

The findings of the present study showed that more than half of the participants were having knowledge and skill deficit about insulin therapy use, which is significantly associated with poor glycemic control (OR = 3.60, P = 0.004). Similarly, the study in Pakistan [22] and Netherlands [33] reported that inadequate health literacy contributed to the disproportionate burden of diabetes-related problems among disadvantaged populations. Possible reasons are time constraint, lack of adequate human power, and most importantly lack of appropriate guidelines and diabetes education for both healthcare providers and patients.

Adherence to medication, diet, exercise and self care improves diabetes management and avoids long term complications. In the present study participants' adherence to insulin regimen, self-care and life style didn't show significant difference (P = 0.509) between good and poor glycemic control, a finding which is in variance with findings from Jordan [17] and Ireland [34] that showed non-adherence to diabetes self-care management behaviours was associated with poor glycemic control. Possible reason for non-adherence in the present study could be inadequate consultation or diabetic education time during refilling of the prescription, and lower educational and health literacy levels of participants. However, adherence to diabetic meal plan to eat more vegetables and fruit (48.2%) in daily meal was significantly associated with glycemic control (P = 0.001) and it was one of the independent predictor of glycemic control (AOR = 0.35, P = 0.005) and the same finding has been reported as effective approach to improve glycemic control among Chinese diabetic patients [35].

A good consultation on regular basis can be vitally important in helping diabetic patients to understand the importance of adherence to medication, exercise, diet and over all life style management. Consultation is more than just prescribing treatment. In the present study, 84% of patients lasted for less than 10 minutes with physician as a consultation with a mean (± SD) of 8.03±2.67 minutes. An observational study conducted in Saudi Arabia [3] showed that the average diabetes consultation with a general practitioner lasted 10 min. This finding implies a belief that there is scope for improving the self-management skills of patients given sufficient time. Indeed, patients' self-management skills and clinical course do improve greatly in response to structured education.

Vials of insulin not in use should be refrigerated. Extreme temperatures (<2 or >25°C) and excess agitation should be avoided to prevent loss of potency, clumping, frosting, or precipitation. Specific storage guidelines provided by the manufacturer should be followed. Insulin in use may be kept at room temperature for a while prior to injection to limit local irritation at the injection site, which may occur when cold insulin is injected. In the present study, the storage condition of insulin was inappropriate for majority of the participants despite 68% of patients were getting insulin for more than or equal to 2 months, which might contribute to instability of the insulin preparation when kept in inappropriate storage conditions over long period of time, and could result in poor glycemic control. A study conducted in India [36] showed similar reports. However, when adequate storage cannot be assured at cool temperatures, insulin vials may be used within three weeks of opening [37], which was not practiced among study participants of the present study due to poor socio-economic status.

This study was the first study conducted at Jimma University Hospital to determine factors associated with glycemic control among insulin treated diabetic patients but has some limitations. Being cross-sectional study is one limitation, where better relationship between glycemic control and different potential factors affecting it progressively cannot be well established, so a longitudinal study is needed to assess the relationship over time. Secondly, the subjective nature of the self-reported response for some items may be limited by recall bias. Thirdly, lack of haemoglobin A1C [38] level for diabetic patients' outcome evaluation in stable conditions might influence the findings of this study, thus findings could not be generalized beyond this study site.

## Conclusions

In conclusion, the proportion of patients with poor glycemic control was considerably high (81.7%), which is higher than reports from many countries. The findings obtained from multivariate logistic regression analysis suggest that poor glycemic control was associated with higher body weight, lower total daily dose, total daily dose variation without evidence of blood glucose level, knowledge deficit about signs and symptoms of hyperglycemia, and poor adherence to dietary plan. Hence, appropriate management of patients focusing on the relevant associated factors and independent predictors identified for poor glycemic control, which are modifiable factors, remains the mainstay to maintain good glycemic control and improve quality of life.
